# Probing the relevance of the accelerated aging mouse line SAMP8 as a model for certain types of neuropsychiatric symptoms in dementia

**DOI:** 10.3389/fpsyt.2023.1054163

**Published:** 2023-02-21

**Authors:** Giorgio Bergamini, Helene Massinet, Aaron Hart, Sean Durkin, Gabin Pierlot, Michel Alexander Steiner

**Affiliations:** ^1^CNS Pharmacology and Drug Discovery, Idorsia Pharmaceuticals Ltd., Allschwil, Switzerland; ^2^Scientific Computing Drug Discovery, Idorsia Pharmaceuticals Ltd., Allschwil, Switzerland

**Keywords:** dementia, agitation, aggression, sociability, SAMP8 mice, deep learning, aging

## Abstract

**Introduction:**

People with dementia (PwD) often present with neuropsychiatric symptoms (NPS). NPS are of substantial burden to the patients, and current treatment options are unsatisfactory. Investigators searching for novel medications need animal models that present disease-relevant phenotypes and can be used for drug screening. The Senescence Accelerated Mouse-Prone 8 (SAMP8) strain shows an accelerated aging phenotype associated with neurodegeneration and cognitive decline. Its behavioural phenotype in relation to NPS has not yet been thoroughly investigated. Physical and verbal aggression in reaction to the external environment (e.g., interaction with the caregiver) is one of the most prevalent and debilitating NPS occurring in PwD. Reactive aggression can be studied in male mice using the Resident-Intruder (R-I) test. SAMP8 mice are known to be more aggressive than the Senescence Accelerated Mouse-Resistant 1 (SAMR1) control strain at specific ages, but the development of the aggressive phenotype over time, is still unknown.

**Methods:**

In our study, we performed a longitudinal, within-subject, assessment of aggressive behaviour of male SAMP8 and SAMR1 mice at 4, 5, 6 and 7 months of age. Aggressive behaviour from video recordings of the R-I sessions was analysed using an in-house developed behaviour recognition software.

**Results:**

SAMP8 mice were more aggressive relative to SAMR1 mice starting at 5 months of age, and the phenotype was still present at 7 months of age. Treatment with risperidone (an antipsychotic frequently used to treat agitation in clinical practice) reduced aggression in both strains. In a three-chamber social interaction test, SAMP8 mice also interacted more fervently with male mice than SAMR1, possibly because of their aggression-seeking phenotype. They did not show any social withdrawal.

**Discussion:**

Our data support the notion that SAMP8 mice might be a useful preclinical tool to identify novel treatment options for CNS disorders associated with raised levels of reactive aggression such as dementia.

## Introduction

People with dementia (PwD) feature a progressive decline in cognition, which co-occurs with several neuropsychiatric symptoms (NPS). NPS pose a major burden for the caregivers and are associated with a worse course of the disease (1). One of the most frequent and pervasive NPS in PwD is agitation; it is characterized by excessive motor activity and reactive verbal/physical aggression, and it is associated with signs of emotional distress (2). From a neuropsychological perspective, agitation has been proposed to arise from an exaggerated response to emotionally salient stimuli (e.g., interaction with caregivers) (3). Social disturbances are also common in PwD, and they may be expressed as socially inappropriate behaviors (4) or as social withdrawal (5).

Most rodent models for dementia were constructed by the over-expression of transgenes that express certain mutated proteins. For example, mouse models for Alzheimer’s disease (AD) over-express specific mutations [e.g., in the amyloid precursor protein (APP) and presenilins (PS)] that are known to induce early-onset familial cases of AD (6). Although these models present age-dependent, AD-related neuropathology leading to cognitive dysfunction, and behavioral disturbances of relevance for NPS [for review see: (7)], they do not recapitulate the chronological sequalae of processes that lead to the late-onset sporadic forms of AD. A model that has been proposed to allow a better investigation of the age-related changes leading to dementia is the Senescence Accelerated Mouse-Prone 8 (SAMP8) mouse (8).

The SAMP8 model is a non-transgenic mouse line with spontaneously occurring gene mutations that displays a phenotype of accelerated aging. In the early 1970’s, researchers at Kyoto University became aware that some of the progeny of the inbred AKR/J colony exhibited a moderate-to-severe degree of accelerated aging (e.g., reduced lifespan) (9, 10). These mice were further bred to yield several specific lines of senescence-accelerated prone (SAMP) mice, along with several lines of senescence resistant (SAMR) mice (showing normal aging) (9), which can be used as age-matched controls. All SAMP lines share common features of rapid advancement of senescence, but each line shows specific characteristics (9). The SAMP8 line models features of age-dependent CNS dysfunction (11) including progressive neuropathological changes and cognitive decline [for review see: (10)]. SAMP8 mice demonstrate neuronal loss (12) and microgliosis (13, 14) starting at 2 months of age, followed by progressive deposition of Aβ (15) starting at 6 months of age, and hyperphosphorylation of tau starting at 5 months of age (16) [for review see: (9)]. Their cognitive deficits manifest as an accelerated deterioration in spatial learning and memory (17) and aversive/appetitive learning (18, 19). Reports on potential NPS-relevant behavioral changes in SAMP8 mice are still sparse: SAMP8 appear to show certain kinds of anxiety-like behavior (20), altered social behavior (21), higher aggressive behavior (22–24) and altered sleep-wake rhythm (25). To the best of our knowledge, no longitudinal characterization and pharmacological validation of agitation-relevant aggressive behavior has been performed in this mouse strain yet. We assessed the reactive aggressive behavior of SAMP8 and control SAMR1 mice in the resident-intruder (R-I) test at 4, 5, 6, and 7 months of age using an in-house developed behavior recognition software, that was trained to recognize aggressive attacks from video recordings. At 7 months of age, we also tested the efficacy of the atypical antipsychotic risperidone (one of the most frequently used drugs to treat agitation in PwD) in reducing aggression in both strains. To probe the relevance of SAMP8 mice for social disturbances in PwD, we measured their social behavior at 7 months of age using the three-chamber social interaction test.

## Materials and methods

### Animals

Senescence Accelerated Mouse-Prone 8/TaHsd (SAMP8) male mice and the corresponding control SAMR1/TaHsd (SAMR1) male mice were purchased from Envigo (The Netherlands). Young, adult male C57BL/6J mice, purchased from Janvier (Le Genest-Saint-Isle, France), were used as intruders in the R-I test and as social stimuli in the three-chamber test. All mice were maintained at the animal facility of Idorsia under standard lab conditions (temperature 20 ± 2°C, relative humidity 55–70%, food and water *ad libitum*) under an inverted 12 h light–dark cycle (lights off 08:00 A.M. to 08:00 P.M.) to be able to perform the behavioral assessments under red-light conditions during the dark phase of the light-dark cycle when nocturnal animals are naturally active. Throughout the study, all mice were provided with enriching material in the home-cage: red, transparent, plastic houses (Techniplast; Buguggiate, Italy), nesting material, and wood sticks; enriching material was only briefly removed during the R-I test so as not to interfere with the video recording of aggressive behavior and to remove possibilities for the intruder mouse to hide from the aggressor. SAMP8 mice were single-housed upon arrival at Idorsia at 7–8 weeks of age to avoid bite wounds between cage mates due to the increased aggressiveness of this strain. SAMR1 mice were treated the same way not to introduce bias in the controls due to different environmental conditions. C57BL/6J mice were group-housed (up to 4 per cage). Mice were kept in individually ventilated cages (IVCs) (GM500, Techniplast).

### Resident-intruder (R-I) test

The R-I test allows the assessment of reactive aggression. It is based on the observation that adult, male, resident mice display reactive aggressive behavior toward unfamiliar male, intruder mice placed in their home-cage (26). The R-I test consists of a confrontation between a single-housed SAMP8 or SAMR1 male mouse (resident) and a previously group-housed C57BL/6J male mouse (intruder). The C57BL/6J intruder mouse is placed in the resident mouse’s home cage, and both mice can freely interact for 10 min. At completion of the 10-min interaction, the intruder mouse is returned to its own home cage. Each test session was video recorded from the top using IP cameras (Axis ML1135L), and quantification of aggressive behavior (i.e., physical attacks, involving direct contact) was performed offline using an in-house developed software (see details further below). The calculated variable was the cumulative duration of attacks during the 10 min of testing. All R-I tests were performed at the beginning of the dark, active phase, under red-light conditions because aggression levels in mice show a circadian rhythm peaking during the dark phase (27). SAMP8 or SAMR1 mice were tested in the R-I test at 4, 5, 6, and 7 months of age. Before starting the first R-I test session (at 4 months of age), SAMP8 and SAMR1 mice were exposed to 3 preparatory R-I sessions to familiarize them to the procedure and to allow stable aggressive behavior to manifest. Once stable aggression was established this way, only a single (at months 5 and 6) or two (at month 7; because of the long duration of the cross-over experiment at this age; see below) additional “reminder” R-I sessions were deemed necessary before the actual testing at the respective ages to re-establish the prior “aggression baseline.” The C57BL/6J interaction partners of the resident mice were changed after each R-I session to avoid habituation/sensitization. At 6 months of age, mice were assessed in the R-I test and treated with vehicle and a small molecule proprietary to Idorsia, using a cross-over design. Similarly, at 7 months, mice were assessed in the R-I test and treated, using a cross-over design, with vehicle, risperidone and with two small molecules proprietary to Idorsia. However, due to the confidential nature of the Idorsia compounds, for the test at 6 months we only report here the data for the session with vehicle, and for the test at 7 months we only report data for the vehicle and risperidone session.

### Drug treatment

Risperidone (R3030, Sigma-Aldrich Chemie GmbH, Buchs, Switzerland) was administered at a dose of 0.2 mg/5 ml/kg *per os*, in Tween 80 0.3%/water. Vehicle consisted of methyl cellulose 0.5%/0.5% Tween 80 (10 ml/kg, *per os*). Treatments were administered 1h before R-I testing using a Latin-square cross-over design: all mice received both treatments in a counterbalanced manner (with 3–4 days between sessions).

### Three-chamber social interaction test (TCT)

The TCT was performed using a sociability cage (Noldus, The Netherlands) made of a square plastic arena (40.5 × 60 × 22 cm) divided in three compartments connected by removable doors. Transparent wired cylinders (ø:10 cm; height: 19 cm) were placed in each of the two lateral compartments and were either empty or contained a social or non-social stimulus; the cylinders allowed the test mouse to explore a stimulus mouse without any physical contact while allowing visual, auditory, and olfactory interactions. The sociability cage was placed in a sound-attenuating cabinet, and an IP camera (Axis ML1135L) was used for video recording the tests (from the top view).

The TCT test comprised 4 phases:

- Habituation: test mice (SAMP8 and SAMR1) were placed into the center compartment with both doors open and one empty cylinder being placed in each of the two lateral compartments. Mice could explore the apparatus for 5 min.

- Sociability (Test 1): test mice (SAMP8 and SAMR1) were placed into the center compartment with both doors open. A cylinder containing a male mouse (Stimulus#1; C57BL/6J, 10 weeks younger than the test mouse) was placed in one lateral compartment, a cylinder containing an inanimate object (i.e., a table tennis ball) in the other one. Mice could explore the apparatus for 10 min.

- Short-term memory (Test 2): 15 min after the end of Test 1 during which time the test mice (SAMP8 and SAMR1) had been placed back in their home cages, they were again positioned in the center compartment with both doors open and allowed to explore the apparatus for 10 min. A cylinder containing the familiar male mouse (i.e., Stimulus#1) was placed in one lateral compartment, a cylinder containing an unfamiliar C57BL/6J male mouse (Stimulus#2) in the other.

- Long-term memory (Test 3): 5 h after the end of Test 1 during which time the test mice (SAMP8 and SAMR1) had been placed back in their home cages, they were again positioned in the center compartment with both doors open and allowed to explore the apparatus for 10 min. A cylinder containing the familiar male mouse (i.e., Stimulus#1) was placed in one lateral compartment, a different, unfamiliar male C57BL/6J mouse (Stimulus#3) was placed in the other one.

The allocation of the two cylinders containing stimulus mice or the object was counter-balanced across subjects. Ethovision software (Noldus, The Netherlands) was used to track the position of three body points of the test mice (nose, body center, tail base). Time spent in proximity (i.e., within 2.5 cm) of the cylinders (using the nose point as a reference) in each of the three test phases was measured.

### Behavior recognition software

An in-house developed computer vision/machine learning (CV/ML)-based software was used to quantify aggressive behavior in the R-I test. Several ML models have been developed in recent years to automate the quantification of aggressive behaviors in rodents (28). Some of this ML software rely on the estimation of the animals’ pose (i.e., location of body parts in space) to classify behaviors, while other ML models are trained to detect the occurrence of specific behaviors without the use of animal’s poses. In pose-free tools, the ML model learns to recognize whether a behavior is present in a specific video frame, thus allowing its quantification (e.g., number of events, cumulative duration in seconds, sequence of behavior) from video recordings of behavioral tests.

In our pose-free ML software, video frames from R-I test videos were pre-processed first by grayscale conversion, followed by rebuilding of the channels not as red, green, and blue color channels (RGB) but as the difference between the current frame and previous frames in the video.

The ML model was implemented as a binary classifier attached via a dense layer to a small convolutional neural network input component. For training, we used a series of R-I test video captures that had previously been identified as containing events of aggression (i.e., physical attacks from one white-coated mouse toward a dark-coated mouse). The model was trained repeatedly on 16 R-I test videos (each 10 min-long) with a single video held out in each run, approximating a Leave One Out cross validation strategy.

The model was built and trained using Keras + Tensorflow (Version 2.2). Following inference by the ML model, a moving average and threshold-based peak detection method was used to bound detected aggressive events as closely as possible to the human analysis. Model performances were measured at a receiver operating characteristic (ROC) value of 0.94 on our validation data.

### Statistical analysis

Data were presented individually and/or as mean and standard deviation (SD). Data for the R-I test (i.e., duration of attacks) at 4, 5, and 6 months of age were analyzed using Welch Two Sample *T*-tests, and data for the test at 7 months of age using two-way repeated measure ANOVA (2-way RM ANOVA) followed by Tukey’s HSD (honestly significant difference) *post-hoc* test. Before analysis, data for 5, 6, and 7 months of age were log transformed, to meet the normality assumption; in case of data points equaling to 0, data were log transformed and a constant was added (i.e., the square root of the first quartile divided by the third quartile). For the TCT, locomotion was analyzed using unpaired t-tests, while the time spent in proximity with the object- or stimulus-containing cylinders was analyzed using 2-way RM ANOVA followed by Sidak’s multiple comparison *post-hoc* test. The threshold for statistical significance was set at *p* < 0.05. Statistical analysis was performed using GraphPad Prism 8 software (GraphPad Software, Inc., San Diego, CA, United States) or R (v 4.2.1).

## Results

### Reactive aggressive behavior

**FIGURE 1:**
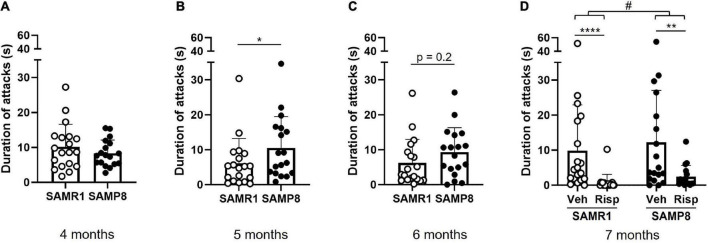
Assessment of aggressive behavior in SAMP8 and SAMR1 in the resident-intruder (R-I) test. Mice were tested monthly between 4 and 7 months of age **(A–D)**. At 7 months **(D)**, using a cross-over design, both SAMP8 and SAMR1 mice were treated with vehicle and risperidone before testing. Duration of attacks was measured from video recordings using an in-house developed ML software. Data are presented individually (scatter) and as means (bar) + standard deviation (error bars). Sample size: *N* = 17-19/group. Sample size: at 4, 5, and 6 months: N = 19 SAMR1, N = 18 SAMP8; at 7 months: N = 19 SAMR1, N = 17(Risp)/18(Veh) SAMP8. *p < 0.05 Welch Two Sample *t*-test; **p < 0.01, ****p < 0.0001, Tukey’s HSD test following two-way RM ANOVA; ^#^*p* < 0.05 main effect of Genotype (two-way RM ANOVA).

In comparison to SAMR1, SAMP8 mice increased their number of attacks with increasing age (Figure 1). While SAMP8 showed a similar level of aggressivity as SAMR1 at 4 months of age [*t*(29.3) = −1.04, *p* = 0.31] (Figure 1A), they were more aggressive at 5 [*t*(33.6) = 2.1, *p* = 0.045] (Figure 1B) and 7 months of age [genotype: *F*(1,68) = 6.0, *p* = 0.017] (Figure 1D); at 6 months of age there was no statistically significant difference [*t*(33.9) = 1.19, *p* = 0.24] (Figure 1C). At 7 months of age, risperidone reduced aggressive behavior [treatment: *F*(1,68) = 39.5, *p* < 0.0001] in both SAMR1 (*p* < 0.0001; *post-hoc* test) and SAMP8 (*p* = 0.002) (Figure 1D).

### Sociability and social memory

**FIGURE 2:**
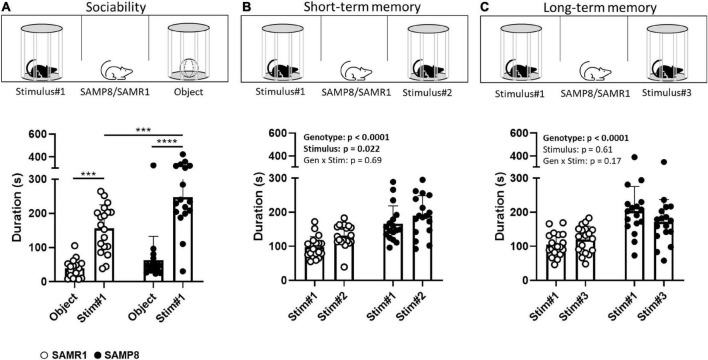
Assessment of sociability and social recognition in SAMP8 and SAMR1 in the Three-Chamber-Test (TCT). At 7 months of age, SAMP8 and SAMR1 were assessed for sociability **(A)** and for short- **(B)** and long-term **(C)** social recognition memory. For each of the three test phases, time spent in proximity with the stimulus-containing cylinders was measured, as an index for stimulus exploration. Data are presented individually (scatter) and as means (bar) + standard deviation (error bars). *N* = 19 SAMR1 and *N* = 17 SAMP8. ***p < 0.001, ****p < 0.0001, Sidak’s *post-hoc* test following two-way RM ANOVA; for clarity reasons, in panels **(B,C)** we describe the main RM ANOVA effects.

During the sociability phase of the TST, as expected, both SAMP8 and SAMR1 mice spent more time exploring the Stimulus#1 mouse relative to the inanimate object [stimulus: *F*(1,34) = 56.79, *p* < 0.0001; Figure 2A]. Interestingly, SAMP8 mice spent more time exploring the Stimulus#1 mouse relative to SAMR1 (*p* = 0.0003, *post-hoc* test following ANOVA) while both strains did not differ in the amount of time spent exploring the object (*p* = 0.51). Locomotion did also not differ between the two strains [*t*(25.5) = 0.15, *p* = 0.88; Table 1].

**TABLE 1:** Locomotion of SAMR1 and SAMP8 during the Three Chamber Test (TCT).

	Test 1			Test 2			Test 3		
SAMR1	3,545	±	478	3,561	±	574	3,577	±	570
SAMP8	3,512	±	802	3,340	±	756	3,060	±	694
*P*-value	0.88			0.33			0.02		

Total locomotion for each of the three test phases of the TCT (test 1/sociability – 10 min; test 2/short-term memory – 10 min; and test 3/long-term memory – 10 min) is presented. Data are presented as means and standard deviation (in centimetres). *P*-values were calculated using individual *t*-tests for each phase.

During the short-term memory phase of the TST, interaction with the unfamiliar mouse (Stimulus#2) was preferred by both SAMP8 and SAMR1, relative to the familiar (Stimulus#1) one [stimulus: *F*(1,34) = 5.803, *p* = 0.0216; Figure 2B], indicative of social recognition memory. There was no genotype × stimulus interaction [*F*(1,34) = 0.17, *p* = 0.69].

In line with the phenotype already observed in the sociability phase of the TST, SAMP8 mice interacted overall longer with their social interaction partners (the familiar and unfamiliar mouse) than the SAMR1 mice [genotype: *F*(1,34) = 52.23, *p* < 0.0001], while locomotion was not different [*t*(29.7) = 0.98, *p* = 0.33; Table 1].

During the long-term memory phase, we did not observe any difference between the time spent with the familiar (Stimulus#1) or unfamiliar (Stimulus#3) mouse for either the SAMP8 or SAMR1 strain indicating that both failed to show any long-term social recognition memory in our setup [stimulus: *F*(1,34) = 0.27, *p* = 0.61; genotype x stimulus: *F*(1,34) = 1.94, *p* = 0.17; Figure 2C]. Again, social interaction overall was higher in the SAMP8 mice in comparison to the SAMR1 [genotype: *F*(1,34) = 96.47, *p* < 0.0001]. This time, SAMP8 moved slightly less during the test than SAMR1 [*t*(31.1) = 2.42, *p* = 0.021; Table 1].

## Discussion

Rodent models can support preclinical research for the identification of novel symptomatic treatments for NPS such as aggression/agitation in PwD. In this study we have used the SAMP8 mouse strain, which recapitulates many of the pathological events occurring during the development of age-related dementia **(8)**. To assess its relevance for studying NPS-related behavioral disturbances, we performed a longitudinal characterization of reactive aggressive behavior (using the R-I test) and of social functions (using the TCT). SAMP8 mice were assessed between the 4th and 7th month of age, a timeframe where many of the neuropathological processes have already started (e.g., neuronal loss, microgliosis) or are starting to build up (e.g., Aβ deposition and hyperphosphorylation of tau) **(9)**, and where cognitive impairment is already present **(13, 29, 30)**. Using the R-I test, we have shown that SAMP8 develop an age-dependent phenotype characterized by higher aggression levels that starts at 5 months of age, and that was observed until 7 months of age, and that is susceptible to treatment with the atypical antipsychotic risperidone, a drug that is used in the clinic to control agitation/aggression in PwD. Another study recently showed that the higher aggression levels of SAMP8 mice could still be observed at 12 months of age (24). This age-dependent increase in reactive aggressive behavior in SAMP8 contrasts with the progressive decline in aggressivity observed in another, transgenic, model of AD-related pathology [i.e., APPswe mice (31)]. This suggests that SAMP8 mice, relative to other models of neurodegeneration and dementia, might better recapitulate the agitation/aggression-relevant behavioral neuropsychiatric disturbances occurring with increasing age and progressing brain pathology. Interestingly, besides risperidone, shown in our study, vafidemstat {a KDM1A and MAOB inhibitor, currently in clinical development for borderline personality disorder and the associated agitation/aggression symptoms [NCT04932291 (32)]}, also attenuated aggressive behaviors in 6 to 7 months old SAMP8 mice **(23)**. These data support the notion that the SAMP8 mouse strain might be a useful preclinical tool to identify novel treatment options for neuropsychiatric disorders associated with raised levels of aggression.

In our experiments the difference in aggressivity between SAMP8 and SAMR1 mice was lower compared to that reported in other studies (23, 24). Several experimental differences apply. First, we have performed the R-I test during the first half of the active phase of the light-dark cycle, while other studies assessed aggression during the light phase (23, 24). However, aggression levels of mice during their active phase are generally higher than during the light phase and not lower (27). Second, contrary to other studies that assessed aggression of SAMP8 at a single age, our study involved repeated assessments. Repetitive reactive aggressive encounter between male mice does not usually lead to habituation and reduction of aggressive behavior of the dominant mouse, but rather, increases the likelihood of expressing the dominant role in future aggressive encounters (33). Therefore, it is unlikely that repeated testing was the reason for the lower aggression levels observed in our hands, also in light of the rather constant duration of attacks of SAMP8 mice during the R-I test between months 5 and 7. Third, we assessed aggressive behavior using an in-house developed ML program. As mentioned in the Methods section, the ROC value of our ML model was 0.94 on validation videos; while this is nominally a good result, aggressive events in the R-I test are relatively rare from a numerical point of view (e.g., less than 1% of the data collected contained examples of mice in conflict). Given these preconditions, setting the threshold of the ML model to maximize its specificity (i.e., avoiding false positives) led to a reduced sensitivity in detecting the events of aggression (i.e., higher false negatives), and thus to an underestimation of aggression events. In addition, the ML tool was trained to score as aggression only events that included a physical attack from the resident to the intruder, while other studies have included in their behavioral scoring also other types of aggressive behaviors (e.g., lateral threats, offensive uprights, “keep-down” behaviors). Despite these limitations, the ML approach bears the clear advantage of enabling a much higher throughput in terms of analysis (e.g., several R-I test videos can be scored in parallel in a very short amount of time, especially if compared to manual analysis) and reduces the potential errors associated with manual analysis such as interpersonal differences in defining what is interpreted as “aggression” or not.

To assess the relevance of SAMP8 mice for the disturbances of social behavior present in PwD, we also tested the mice in the TCT. At 7 months of age, SAMP8 mice showed a higher exploration time toward the social stimulus relative to the unanimated object (indicative of a preserved social preference), and a greater amount of time spent exploring the social stimulus relative to SAMR1 mice. Companys-Alemnany et al. (21) also described the preserved preference for the social stimulus in 6 months old SAMP8 male mice, albeit combined with reduced social interest (i.e., less time spent sniffing the social stimulus). Conversely, older (12 months old) SAMP8 mice showed lower preference for the social stimulus (24). Overall, during the three phases of the TCT, in our hands SAMP8 mice showed a higher exploration time toward the social stimuli relative to SAMR1 mice. This increased social interest does not reflect the symptoms of social withdrawal often observed in PwD (5), but it co-occurred with an aggressive phenotype. It is possible that the higher aggressivity of SAMP8 mice, measured in the R-I test, would be expressed as increased exploration toward the stimulus mice in the TCT. Indeed, it must be considered that the TCT was performed after several sessions of R-I testing, where SAMP8 mice could repeatedly experience the status of social dominance over the intruder (C57BL/6) mice. Given that the experience of previous “wins” during dyadic confrontations increases the motivation to seek that experience again (28, 33), once tested in the TCT, SAMP8 mice might have been more motivated to contact the stimulus (C57BL/6) mice to reiterate the occurrence of the dominance status experienced during the previous R-I tests.

Although other studies have shown that 7-months old SAMP8 mice have cognitive impairment [e.g., impairment of spatial memory in the Morris water maze (34)], short-term social recognition memory was apparently still preserved at 7 months of age in both strains, while we could not measure the formation of long-term social recognition memory using the chosen testing conditions, in either strain. It is possible that the higher propensity of SAMP8 mice in exploring the social stimuli might have occluded the detection of a cognitive impairment in this test; alternatively, cognitive impairment of SAMP8 mice at 7 months of age might not yet include social memory or might not yet have been so profound as to be detectable using the TCT. In sum, in our investigation, SAMP8 mice do not show behavioral impairment of relevance for the social withdrawal that is present in PwD.

Overall, our study supports the notion that the SAMP8 mouse strain represents an interesting preclinical model for age-related reactive aggression, which is a specific type of NPS occurring in PwD. SAMP8 mice can, thus, be used to assist the development of new symptomatic drug treatments for aggression in neuropsychiatric disorders.

## Data availability statement

The original contributions presented in this study are included in the article, further inquiries can be directed to the corresponding author.

## Ethics statement

Experimental procedures were approved by the Basel-Landschaft Veterinary Office and adhered to Swiss federal regulations on animal experimentation.

## Author contributions

GB, AH, and MS: conceptualization. GB and AH: writing – original draft preparation. GB, HM, SD, AH, GP, and MS: writing – review and editing. GB, HM, SD, AH, and GP: investigation and formal analysis. AH: software. MS: supervision. All authors contributed to the article and approved the submitted version.
